# 

**Published:** 1871-07

**Authors:** 


### CONTENTS.

COMMUNICATIONS.

Dental Physiology ..... 281
Professional Fidelity ..... 290
Phrenology and Dentistry ..... 295
Address read before the Northern Ohio Dental Association ..... 301
A Case in Practice ..... 304
Professor Tyndall on the Chemistry of Light—New Experiments and Conclusions ..... 306
Heavy Foils ..... 308

PROCEEDINGS OF SOCIETIES.

Northern Ohio Dental Association ..... 311

EDITORIAL.

The Physiology of Thought ..... 319
Dental Organization in Kansas ..... 321
Surgery that is Surgery ..... 321
The Pneumatic Engine-Improvement ..... 322
Insanity in Woman ..... 323

